# Applications of Catalytic Nanomaterials in Energy and Environment

**DOI:** 10.3390/molecules28104000

**Published:** 2023-05-10

**Authors:** Hongda Li, Shuai Jian, Mohammed Baalousha

**Affiliations:** 1 Liuzhou Key Laboratory for New Energy Vehicle Power Lithium Battery, School of Electronic Engineering, Guangxi University of Science and Technology, Liuzhou 545006, China; 2 Center for Environmental Nanoscience and Risk, Department of Environmental Health Sciences, Arnold School of Public Health, University of South Carolina, 921 Assembly Street, Columbia, SC 29208, USA

Nanotechnology is a crucial technology for the development of science and technology. Nanomaterials, constituting a key building block of nanotechnology, are solid objects with at least one dimension between 1 and 100 nm. Nanomaterials include nanoparticles, nanosheets, nanofibers (rods, tubes, etc.), nanodots, etc. Due to their small size, nanomaterials possess unique and novel properties and functions such as increased specific surface area and reactivity, and quantum confinement. Therefore, nanomaterials have a wide range of applications, including medical diagnosis, material modification, environmental remediation, biotechnology, catalysis and photocatalysis, etc.

Catalysts, substances that increase the rate of a reaction without undergoing permanent chemical changes, play important roles in the chemical industry and the environment. Catalysts have been used in the treatment of fuels such as oil, natural gas, and coal and in the purification of wastewater and industrial waste gases. Catalysts can be classified as homogeneous or heterogeneous catalysts. The former refers to catalysts that exist in the same phase (gas or liquid) as the reactants, whereas the latter refers to catalysts that are not in the same phase as the reactants. Heterogenous catalysis occurs at the interface of two phases, usually with a porous solid catalyst and a liquid or gas reactant. For chemicals to react, their chemical bonds must be changed, and a certain amount of energy is needed to change or break these chemical bonds. The minimum energy threshold required to change chemical bonds is called activation free energy, and a catalyst affects a reaction rate by changing the activation free energy of the chemical reactants. Compared with homogeneous catalysts, heterogeneous catalysts possess higher selectivity and offer better yields. Therefore, heterogeneous catalysts are receiving much more attention than homogeneous catalysts. To improve the efficiency, yield, and purity of catalysts, it is very important to study new catalytic materials or optimize existing catalyst systems.

At present, the research in this field mainly focuses on nano-structured catalysts with special physical and chemical properties. Nanoscale catalysts possess higher surface area and surface energy compared with non-nanoscale catalysts, which ultimately results in higher catalytic activity. Nanocatalysts improve reaction selectivity by reducing the required activation energy and the occurrence of side reactions and improving the recovery of energy consumption. Therefore, nanocatalysts have been widely used in green chemistry, environmental remediation, the efficient conversion of biomass, renewable energy development, and other fields. This Special Issue focuses on the application of nanocatalysts in the field of energy and environment.

In addition to producing clean energy efficiently, nanocatalysts enable the recycling of wastewater, waste gas, and solid waste, which is crucial to solving energy and environmental problems. In recent years, great progress has been made in thermal catalysis, photocatalysis, and electrocatalysis for energy and environmental applications. In particular, pollutant degradation [1,2,3,4,5,6,7], green hydrogen production [8], indoor formaldehyde purification [9], oxygen reduction [10], and other fields [11,12,13] have ushered in major developmental opportunities and important original breakthroughs.

The controllable preparation and structure–activity relationships of nanocatalysts are crucial aspects with respect to overcoming the bottleneck regarding nanocatalysts’ application. The design of nanocatalysts has evolved from the initial macro-strategies, such as component regulation, size control, and morphology tailoring, to the precise regulation of the catalytic center coordination and electronic structures at the subatomic/atomic level. At the same time, the development of advanced nanocatalysts is inseparable from the bold innovations in preparation strategies, the technical innovations in characterization methods, the iterative renewal of the concept of catalysis, and the continuous exploration and re-evaluation of the objective law of the structure–activity relationship.

This Special Issue focuses on energy and the environment, including with respect to the latest research results, experiences, and prospects, and contains eleven original research papers. Most of the studies focus on the synthesis and related mechanisms of photocatalytic nanomaterials. Photocatalysis technology has great potential for solving energy shortages and environmental pollution. Improving the sunlight utilization efficiency and catalytic activity of photocatalytic nanomaterials constitutes the key problem restricting the large-scale application of photocatalytic technology. This Special Issue presents a detailed account of the internal mechanism whereby photocatalytic activity is enhanced via elemental doping [1,2,3], the construction of composite heterogeneous structures [4,5,6,10], the introduction of organic photosensitive materials [8], morphology control [9], and so on. These contributions provide various approaches to the design and establishment of efficient photocatalytic nanomaterials. This Special Issue also presents two works on the use of metal–organic frameworks (MOFs) for efficient ammonia capture [11,12]. Although these two works do not involve catalytically related applications, most of these MOFs have high porosity and good chemical stability. Due to their ability to control pore structure and their large surface area, MOFs have wider application prospects in the catalyst field than other porous materials. The unsaturated metal sites of MOF materials, acting as Lewis acid sites, can be used as a catalytic center. MOFs have been used in the cyanidation reaction, the oxidation reaction of hydrocarbons and alcohols, the esterification reaction, the Diels–Alder reaction, and in other catalytic applications. This Special Issue also includes two review articles summarizing the state of the art in the two important areas of the application of photocatalysis and sonocatalysis for the treatment of organic dye wastewater [7] and the preparation, passivation, and application of two-dimensional black phosphorus in lithium-ion batteries [13]. These review articles cover a wide range of topics, including the preparation methods, characterization techniques of energy and environmental catalytic materials, and practical applications of nanocatalysts in the field of photo/electrochemistry. It is hoped that the publication of this Special Issue will advance the field of “catalysis for energy and the environment” and provide the reader with a launch point into this highly innovative area of research.

## Data Availability

Not applicable.
